# Visualization of Inflammation at Early Stage of Lung Cancer in Xenografted Temporally Immunosuppression Rats by Ferrioxamine Magnetic Resonance Imaging

**DOI:** 10.1155/2016/8434308

**Published:** 2016-12-15

**Authors:** Nathupakorn Dechsupa, Chatchanok Udomtanakunchai, Anan Udom-Utraracheva, Dutsadee Suttho, Lionel Pazart, Philippe Humbert, Manuel Garrigos, Samlee Mankhetkorn

**Affiliations:** ^1^Laboratory of Physical Chemistry, Molecular, and Cellular Biology, Center of Excellence for Molecular Imaging, Department of Radiologic Technology, Faculty of Associated Medical Sciences, Chiang Mai University, 50200, Thailand; ^2^Clinical Investigation Center of Besançon, Inserm CICB 1431, University Hospital of Besançon, 2, Place St. Jacques, 25030 Besançon, France; ^3^Cutaneous Engineering and Biology Team, Inserm UMR 1098, University of Bourgogne Franche-Comté, 19 rue Ambroise Paré, Besançon, France; ^4^Department of Structural Biology and Bioenergetics Mechanisms, Commission for Atomic Energy, Institute of Biology and Technologies-Saclay, 91191 Gif-sur-Yvette, France

## Abstract

Physiological responses such as chronic inflammation and angiogenesis could be used as biomarkers for early detection of cancer with noninvasive imaging modalities. The present study reports the application of magnetic resonance imaging instrument to image the binding of ferrioxamine with hemin that allows visualizing the chronic inflammation foci of lung tissue of immunocompromised rats xenografted using small cell lung carcinoma. A low concentration of ferrioxamine (0.05 ± 0.02 *μ*M·kg^−1^ of rat weight) deposited on tissue outside the vasculature was found to diffuse across the capillary walls to the interstitial space and inflammation foci, which provided a clear enhancement of T1-weighted gradient-echo sequence images. Ferrioxamine imaging allowed the determination of inflammatory sites and their localization in 3D fat-suppressed maximum intensity projections. The smallest dimension of foci that can be clearly determined is about 0.1 mm^3^. In concomitant to the* in vivo* imaging, analysis of histological tissue section showed the development of inflammatory sites. This study provides evidence that medical imaging instrument such as MRI scanner allows researchers to correlate images taken with MRI with those using high-resolution microscopy. Moreover, ferrioxamine is a useful molecular probe for determining chronic inflammation particularly at the very early stages of cancer.

## 1. Introduction

Tumor-bearing animals offer a variety of xenograft-based cancer models may greatly help understanding cancer biology, especially for the interactions among cancer cells and normal cells* in vivo* situation. For this purpose the need of tumor-bearing animal models that can be elucidating the behaviour of cancer cells when faced to the host immunity pressure. However, almost all animal models of cancer reported in literatures showed irreversible immunosuppression. For example, the use of total lymphoid irradiation, transgenic mice, or rats [1–5] showed a high take rate of cancer after having been xenografted with human cancer cells. The migration of endothelial cells from surrounding tissues to the point where cancer cells were inoculated was found during two weeks after human cancer cells were implanted into animals. This was followed by the appearance of cancer stromal as well as angiogenesis. These events can be clearly visualized by the immunohistochemical staining of endothelial cells and hematoxylin-eosin staining of red blood cells as well as leucocytes accumulation found outside the blood vessels of the tissue. Then these solid tumors had rapidly grown and could be clearly measured using vernier calipers. In our research groups, the tumor diameter of about 1.5 cm usually obtained after 1 month in athymic nude mice xenografted with MDB-435 human breast cancer cells [5]. It should be noted that the tumor was rapidly increased in size reach to the limit of the animal use guideline; the animals must be terminated. Thus the interaction/behaviour of cancer cells in normal microenvironments in particularly under the pressure of host antibody cannot be detected. Indeed these models are useful for a variety of studies such as in testing drugs or radiation sensitivities and targeting of biomarkers for cancers. There is evidence that the tumor formation in irreversible immunosuppressed animal models takes very short time that will be limitation for investigation of the interaction of cancer cells with normal cells in the microenvironment of normal tissue. Therefore, a temporally immunosuppressed animal model that will not reject human cancer cells at the starting period where afterwards a reversible suppressant can be achieved after withdrawal is crucial for understanding the phenomenon. Kaartinen et al. [6] and Hoogenhout et al. [4] showed that Wistar rats treated with cyclosporin A develop a state of immune suppression that permits the growth of tumor xenografts. It was also demonstrated that in these models there was no alteration in the tumor doubling time or histological morphology of the xenografts in the adapted host when compared to those in the donor tumors. The tumor growth curve showed a pattern of initial growth and a period of stagnation, followed by a steady, but slower growth phase. It should be noted that animals were continuously given cyclosporin A throughout the experimental periods. Since cyclosporin A is a potent reversible immunosuppressive agent, it can facilitate the induction of immunologic tolerance* in vivo* in a variety of animal models. It was shown that the reversible suppressant activity affected both humoral and cellular immunity and did not cause myelosuppression. Its mechanism of action appears to be selective for lymphocytes and may interrupt the necessary cellular signals required for proliferation of alloreactive T-cells [7, 8].

In this study, we prepared temporally immunosuppressed Wistar rats by consecutive intraperitoneal injections of cyclosporin A (200 mg·kg^−1^) for 4 days prior to the human small cell lung carcinoma (GLC4 and GLC4/adr) xenografts. Since the immunosuppression mediated by cyclosporin A is known to be a reversible process and after withdrawing cyclosporin A, the rat's immune system fully recovered within one week [9–12]. In this condition, on one hand the cancer cells might have enough time to depart from their point of origin of xenograft to various organs and prepare the stromal structure for adhering and caused leakage of red blood cells and leucocytes to outside the blood vessels of a tissue following inflammation. On the other hand the GLC4 and CLC4/adr cells themselves are characterized as hypoxic cells and are strong triggers of angiogenesis which is clearly observed by immunohistochemistry of the lung tissue [the results were presented in WMIC 2012]. The results of histological studies revealed various sites of inflammation that were not observed in similar series of experiment performed using athymic nude mice. Therefore, the temporally immune-suppressed animal appears as a very good model that appropriately studies hypoxic and inflammation of microenvironment during the early stages of lung cancer.

It is well accepted that an accumulation of red blood cells and leucocytes outside the blood vessels of a tissue is evidence of an early stage of angiogenesis. The accumulation of red blood cells outside the blood vessels for long time causes an increase in old red blood cells that underwent phagocytosis by the tissue macrophages or an old red blood cell lysis which may lead to a subsequent release of the prooxidant haemoglobin. Then the haemoglobin was oxidized in met-haemoglobin and liberates heme and hemin within tissues [13, 14]. Consequently it induces the tissue to be found in hypoxic microenvironment condition. An increase in iron accumulation in tissue will trigger an immune-inflammatory response and hypoxic microenvironment is known as an early event of cancerous tissue formation [15–18]. Evidence is rapidly accumulating that chronic inflammation may contribute to carcinogenesis through the increase of cell proliferation, angiogenesis, and metastasis in a number of neoplasms, including colorectal carcinoma [19, 20].

In fact hemin is a prominent biomarker of the immune-inflammatory response and hypoxic microenvironment that can be noninvasively visualized by using ferrioxamine magnetic resonance imaging. Ferrioxamine is a stable complex of desferrioxamine (DFO) with ferric ion. Complexation of ferric-DFO was done using stoichiometry one mole of DFO with one mole of ferric ion; *K*_*a*_ of the complexation of ferric with DFO is 10^31^ mole^−2^ [20]. The interaction of DFO and hemin was investigated and the binding constant of DFO–hemin (1 : 1 complex) was calculated to be 10^4^–10^5^ M^−1^ at various temperatures [21]. In this study it is also verified that the ferrioxamine can specifically bind to heme neither without decomplexation nor reduction of ferric ion. Ferrioxamine can enhance the sensitivity of proton relaxation signal detection of a tissue equivalent phantom by 1000-fold higher than without the complex condition [20], indicating that the ferrioxamine MR imaging might be able to determine the number of molecules (<10^−6^ M) of water participating in the excitation and relaxation processes. An administration of ferrioxamine in animal models and the specific interaction of heme-ferrioxamine complex allow investigating the chronic inflammation site original of lung cancer. The smallest dimension of foci that can clearly be visualized is about 0.1 mm^3^ that is sensitive enough to visualize the inflammation and angiogenesis of the early stages of cancer tissue.

## 2. Materials and Methods

### 2.1. Chemicals and Preparation of Solutions

Cyclosporin A was purchased from Novartis. Iron(III) chloride anhydrous (FeCl_3_) was obtained from Fluka; desferrioxamine mesylate, hemin, and Pirarubicin were from Sigma.

### 2.2. Decontamination of Adventitious Metals and Buffer Used

The presence of contaminating metals in reagents can lead to unpredictable change in any additional enhancement of proton signals due to the exchange of contaminating metals with iron, which may affect the rates of proton relaxation. Therefore, in order to remove trace amounts of adventitious metals in water, chelating resin (Chelex 100) was used as previously described [22]. Chelex 100 resin was added to double-distillated water (5 mg/100 mL) and stirred gently for 1 hour. Next, the water was filtered from the resin using filter paper for quantitative analyses (MN 615, Macherey-Nagel).

The buffer solutions were prepared in glassware. The glassware was washed with 10% nitric acid and rinsed three times with metal-free water.

The buffer solution contained 20 mM Hepes buffer supplemented with 132 mM NaCl, 3.5 mM KCl, 0.5 mM MgCl_2_, and 5 mM glucose, pH 7.25.

All other reagents were prepared in disposable plastic wares. The stock solution of 0.01 M ferric ions (in 0.4 M H_2_SO_4_) was prepared extemporaneously. The stock solutions of 0.01 M desferrioxamine were prepared in metal-free water and stored at −20°C. To obtain the desired concentration of desferrioxamine, the stock solution was diluted with a buffer.

The absorption spectra were recorded on an Agilent 8435 spectrophotometer. Experiments were conducted in a 1-cm quartz cuvette containing 2 mL of solution that was continuously stirred. The temperature was kept constant at 25°C using a Peltier temperature control, cell holder model 89090A.

Ferric ion concentration was determined by a spectrophotometric method. The lyophilized products were dissolved in 0.4 M H_2_SO_4_. The organic compounds were eliminated by addition of 0.01 M HCl. The concentration of ferric ion was determined using a molar extinction coefficient at 304 nm equal to 2204 M^−1^·cm^−1^ [23].

### 2.3. Preparation of Ferrioxamine

Ferrioxamine was prepared extemporaneously in Hepes buffer. DFO in an aqueous solution is colorless while ferric ions have a bright yellow color. A reddish color appears immediately after the solution of DFO comes in contact with ferric ions. The solution mixture possessed an absorption spectrum from 350 to 450 nm with a maximum absorbance at 427 nm. The concentration of ferrioxamine was determined using a molar extinction coefficient at 427 nm equal to 1670 ± 200 M^−1^·cm^−1^ [20]. Lu et al. showed that DFO bind on hemin with high affinity [21]. In this study, we had checked that ferrioxamine neither can bind to hemin without releasing nor underwent reduction of ferric ion.

### 2.4. Cell Culture and Cytotoxicity Assay

The human small cell lung cancer, GLC4, and its MRP1-overexpressing GLC4/adr cell lines were routinely cultured in RPMI 1640 medium supplemented with 10% fetal calf serum (Gibco Biocult, Ltd.). The resistant GLC4/adr cells were cultured in the presence of 100 nM doxorubicin until two weeks before the experiments. For the assays, a culture was initiated at a density of 5 × 10^5^ cells·mL^−1^ in order to have cells in the exponential growth phase. The cultured cells were used 24 hours later, when the culture had grown to a density of approximately 8 × 10^5^ cells·mL^−1^. Cell viability was assessed by the Trypan blue exclusion assay. The number of cells was determined using a hemocytometer or by flow cytometry.

The cytotoxicity assay was performed as follows: 5 × 10^4^ cells·mL^−1^ were incubated in the presence of various concentrations of compounds ranging from 0 to 100 *μ*M. The cell viability was determined by 3-(4,5-dimethyl-2-thiazolyl)-2,5-diphenyl-2H-tetrazolium bromide (MTT)-reduction assay. IC_50_ was determined by plotting the percentage of cell growth inhibition versus the concentration: IC_50_ was the concentration of compounds which inhibits cell growth by 50% when measured at 72 hours. A resistance factor (RF) was defined as IC_50_ of resistant cells divided by IC_50_ of the sensitive cells.

### 2.5. Preparation of Small Cell Lung Carcinoma Xenografted Rats with GLC4 and GLC4/Adr Human Cells

A total of 9 male Wistar rats (Outbred: National Laboratory Animal Center, Mahidol University, Thailand) each being 4 weeks of age were housed in sterile isolation unit and fed rodent chow and water* ad libitum* and were treated in accordance with institutional guidelines for animals. Rats were intraperitoneally injected with cyclosporin A (200 mg·kg^−1^) for 4 consecutive days prior to the implantation of GLC4 and GLC4/adr cells. The cancer cells (10^7^ of each cell line in 200 *μ*L DPBS) were subcutaneously implanted at the lower limbs; left site for GLC4; and right site for GLC4/adr cells. The xenografted rats were examined by monitoring the plasma metabolites using a 400 MHz nuclear magnetic resonance spectrometer, for the formation of cancer tissue at the implanted sites, with MR imaging being done every month.

### 2.6. MR Imaging of Ferrioxamine

Rats were anesthetized by intraperitoneal injection with 2 mg·kg^−1^ of Nembutal (OVATION Pharmaceutical Inc., Deerfield, Illinois, USA). The tail was intravenously punctured with a catheter 24 G, and a drip of NSS was administered [5]. The anesthetized rats were placed and fixed in a prone position inside an in-house made bed (to keep at a constant temperature of 25°C) equipped with a head coil. The MR images were performed using a 1.5 T MRI instrument (Achieva, Philips). The coronal and sagittal plan surveys were obtained using the same scout techniques that were applied to both T1w-SE (TR = 535 ms; TE = 10 ms) and T2w-TSE (TR = 2500 ms; TE = 115 ms). The FOV was 279 mm. The ferrioxamine imaging was performed at 20 seconds and then was performed at 2, 4, 6, 15, 45, and 60 minutes after a 50 *μ*mol kg^−1^ injection in the tail vein had been administered.

#### 2.6.1. Analysis of MRI Data and Biodistribution

Signal intensity values at each time point were obtained from regions of interest (ROIs) and were determined using a series of cross section images as a function of time. Signal intensities from three ROIs taken from different sections of each tissue of interest were averaged, and the mean SI was used as a basis for further calculations. The signal from a spin echo sequence is as follows: *S* = *kρ*(1 − *e* ^(−TR/*T*_1_)^) · *e* ^(−TE/*T*_2_)^, where *kρ* is a constant related to magnetization density; TR and TE are the sequence repeat time and echo time, respectively. With respect to the protocol of T1w-imaging, TR ≫ TE, where TE had the lowest value, the signal intensity obtained from the scanning becomes *S* = *kρ*(1 − *e* ^(−TR/*T*_1_)^). The equation can be arranged by replacing the signal intensity (*S*) and *kρ* by *I* and *I*_0_, respectively:

(1) $$\begin{array}{c} I=I_{0}\left(\;1-e^{\left(-TR/T_{1}\right)}\;\right). \end{array}$$

Thus, the density of T1w-images can be written as

(2) $$\begin{array}{c} \text{Density}=\log\frac{I_{0}}{I}=0.3623\frac{TR}{T_{1}}. \end{array}$$

The relationship between relaxation rates and contrast agent concentration can then be predicted by the Solomon-Bloembergen equations [24, 25]:

(3) $$\begin{array}{c} \frac{1}{T_{1}^{\text{postCon}}}=\frac{1}{T_{1}^{\text{preCon}}}+R1\left[\;M\;\right], \end{array}$$

where *R*1 is the longitudinal relaxation being equal to 1.05 ± 0.4 mM^−1^·s^−1^ and [*M*] is the concentration of ferrioxamine. The ferrioxamine enhanced spatial image contrast was measured using the following equation:

(4) $$\begin{array}{c} \frac{{Density}_{postCon}}{0.3623TR}=\frac{{\text{Density}}_{\text{preCon}}}{0.3623TR}+R1\left[\;M\;\right]. \end{array}$$

#### 2.6.2. Tissue Preparation and Immune-Histochemical Analysis

Immediately after sacrificing the rats, organs were harvested, weighed, and cut into small pieces. Fragments were fixed with 4% formalin and processed in paraffin in the usual way, and 5-*μ*m sections were stained with hematoxylin-eosin. Endothelial cells were specifically stained with GSL-1 lectin (Vector Laboratories, Burlingame, CA) in the previously described manner [1]. For each GSL-1-labeled section, five fields are containing exclusively viable tumoral cells. The fields which were indicated by the hematoxylin staining were randomly selected for analysis. The percentage area of endothelial cells was then calculated as a ratio of the labeled area: the total viewed area × 100. Tissue accumulation of iron was determined by Perls' Prussian blue with hematoxylin counterstain method.

## 3. Results

In order to apply ferrioxamine as a molecular probe for MR imaging of lung tissue inflammation, the cytotoxicity of the compound against human small cell lung carcinoma GLC4 and GLC4/adr was performed. DFO alone is moderately toxic against small cell lung carcinoma (GLC4; IC_50_ = 30 ± 6 *μ*M) and its corresponding multidrug resistant cells which overexpressed MRP1-protein (IC_50_ = 50 ± 3 *μ*M) compared with Pirarubicin, (GLC4, IC_50_ = 10 ± 3 nM and GLC4/adr, IC_50_ = 42 ± 5 nM). Ferrioxamine exhibited cytotoxicity against the same cell lines 4 times lesser than those of DFO alone.

### 3.1. Biodistribution of Ferrioxamine in Normal Rats

**Figure 1:**
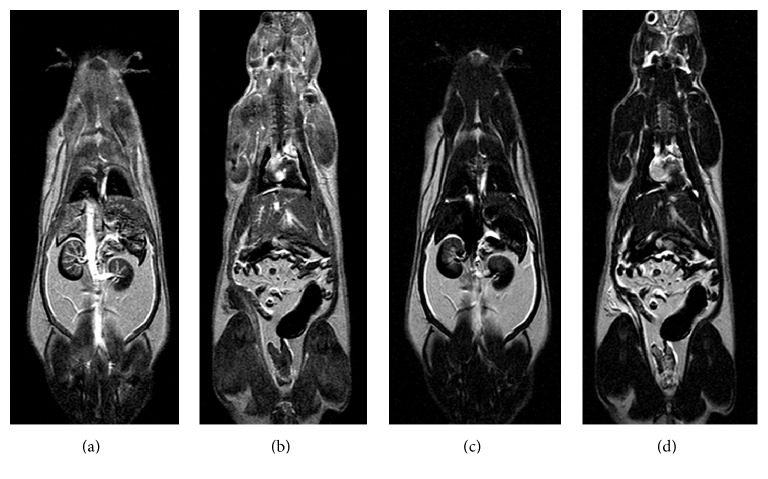
Representative of coronal images of normal rats at 6 minutes after ferrioxamine administration of T1w-SE (a and b) and T2w-TSE (c and d). The anesthetized rats were placed and fixed in a prone position inside an in-house made bed (allowing controlling the temperature at 25°C) compatible with head coil. The section thickness was 2 mm and began from dorsal to ventral. The ferrioxamine imaging was performed at 20 seconds and then 2, 4, 6, 15, 45, and 60 minutes after injection 50 *μ*mol·kg^−1^ in tail vein.

Ferrioxamine at a subtoxic dose of 50 *μ*mol kg^−1^ was intravenously injected to the rats and was sufficient to enhance the image contrast, without inducing any adverted effects during experiments. As can be observed in Figure 1, T1w- and T2w-images of Wistar rats after administering the paramagnetic agent clearly showed an increase in the T1-signal intensity while they showed a decrease in the T2w-signal intensity in various tissues such as the vascular systems and urinary system (including renal cortex and pelvis, ureters and bladder, brains, and muscles). However, no change was observed for the fat tissues. The ferrioxamine should be useful as a T1-contrast agent for MR imaging.

Due to limitation of MRI instrument, the fastest dynamic imaging protocol can performed at 20 seconds and then at one-minute time intervals. The first pass of ferrioxamine to heart and aorta cannot be determined by using dynamic enhance contrast MRI; however this can clearly be determined by CT. In the conditions of experiments, ferrioxamine (bolus of 50 *μ*mole) which was administrated in the tail vein was reached to the heart and ejected via aorta for first pass within 20–22 seconds.

**Figure 2:**
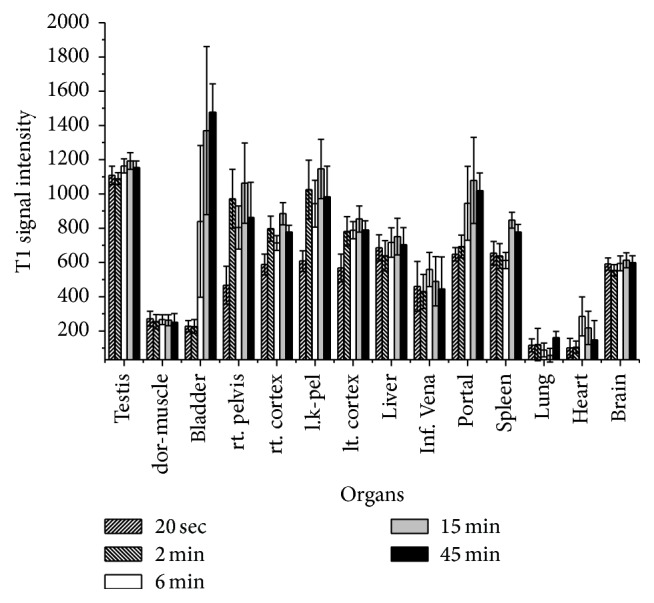
Average pixel density determined by ROI technique of T1w-SE images of cross section of normal rats as a function of time. The ferrioxamine imaging was performed at 20 seconds and then 2, 4, 6, 15, 45, and 60 minutes after injection 50 *μ*mol·kg^−1^ in tail vein. The results were obtained from the series of experiments indicated in Figure 1 and reported as mean ± SD.

**Figure 3:**
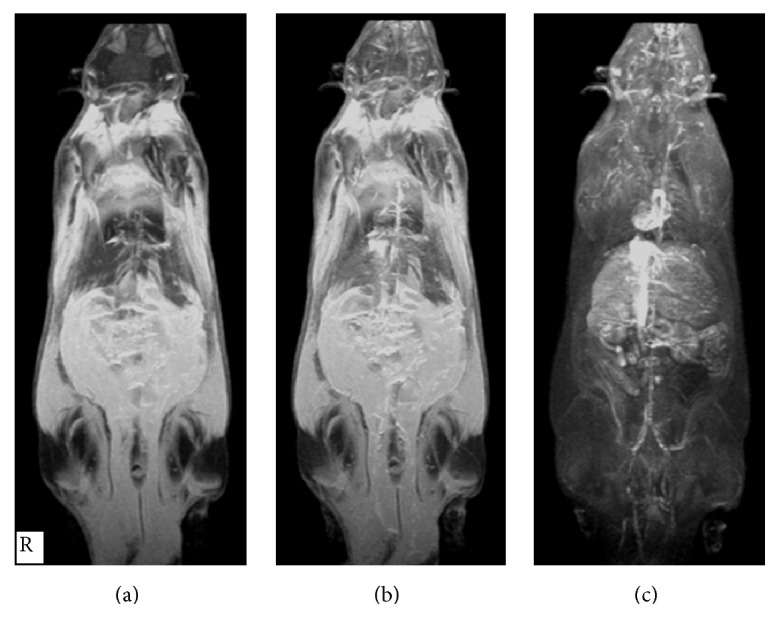
Whole body 3D-MRI (MIP) to show the T1w-SE scout view (a), T1w-SE (b), and T1w fat suppression (c) after injection of ferrioxamine (50 *μ*mol·kg^−1^) at 45 minutes. The anesthetized rats were placed and fixed in a prone position inside an in-house made bed (allowing controlling the temperature at 25°C) compatible with head coil. The section thickness was 2 mm and began from dorsal to ventral.

Figure 2 illustrates the distribution of ferrioxamine accumulated in different organs at various times. The compound reached the kidneys and bladder at 4 and 6 minutes, respectively.  Figure 3 showed that ferrioxamine penetrated across the endothelial cell layer and resides into the tissues. The tissue concentration of ferrioxamine determined by MR imaging technique was 0.02 ± 0.01 *μ*mole·kg^−1^ of rat weight for muscle, 0.01 *μ*mole·kg^−1^ of rat weight for lung, and 0.02 *μ*mole·kg^−1^ of rat weight for brain.

#### 3.1.1. Small Cell Lung Carcinoma Cell Xenografted Rats

**Figure 4:**
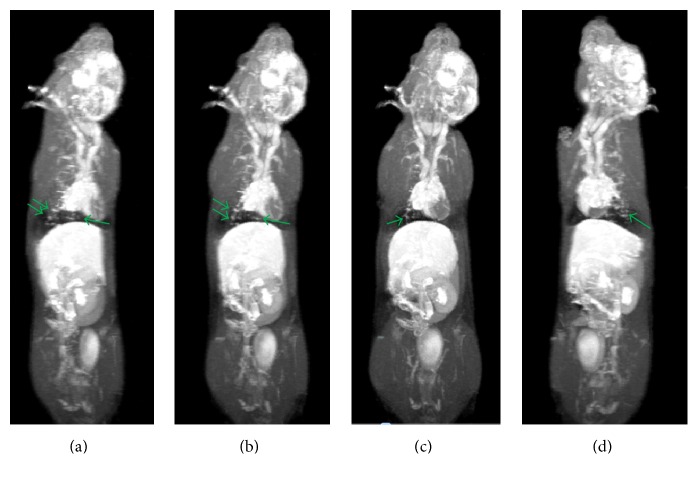
Whole body 3D-MRI (MIP) to show T1w fat suppression after injection of ferrioxamine (50 *μ*mol·kg^−1^) at 45 minutes of a rat after six months xenografted with human GLC4 and GLC4/adr cell in different angle of rotation axis to show bright spots in thoracic cavity enhanced by ferrioxamine (green arrows). The anesthetized rats were placed and fixed in a prone position inside an in-house made bed (allowing controlling the temperature at 25°C) compatible with head coil. The section thickness was 2 mm and began from dorsal to ventral.

**Figure 5:**
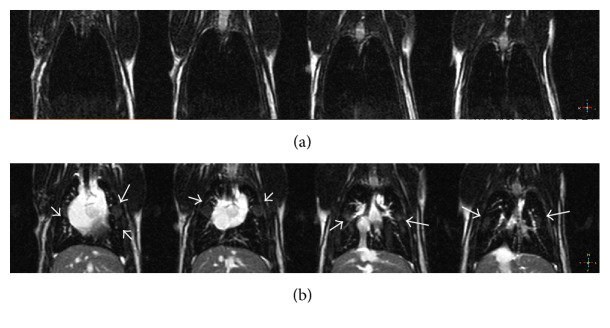
Coronal view of MR chest images using T1w-SE sequence of human GLC4 and GLC4/adr cell xenografted rats before (a) and at 6 minutes after injection of 50 *μ*mol·kg^−1^ (b). White arrows indicated the bright spots corresponding to an accumulation of ferrioxamine at the spots.

T1w-images showed the typical results of biodistribution of ferrioxamine in xenografted rats. The 3D images of whole body of rats can clearly elucidate the tissue distribution pattern and accumulation of ferrioxamine in xenografted rats (Figure 4). The arterial phase of ferrioxamine was recorded within 20 to 40 seconds and then following the venous phase at 6 to 15 minutes and the equilibrium phase at 45 to 60 minutes. As can be seen in Figure 4, the images of xenografted rats (3 months after xenografted) with injection of ferrioxamine compared with those without revealed that there were bright spots distributed throughout the lung and a loss of signal area at the anterior of left ventricle (Figure 5).

It should be noted that these bright spots are very small and the average diameter was approximately 0.6 ± 0.1 mm. The typical enhancement of T1-signal was not found in organs other than lung. These bright spots should correspond to the infiltration of lung tissue indicative of an inflammation which is the early event of cancer. It is very difficult to determine the tissue concentration of ferrioxamine in lung tissue by excluding the bright spots in these series of experiments. But concentration of ferrioxamine of the bright spots of lung tissue was around 0.05 ± 0.02 *μ*mole per kg of rat weight.

#### 3.1.2. Histological Study

**Figure 6:**
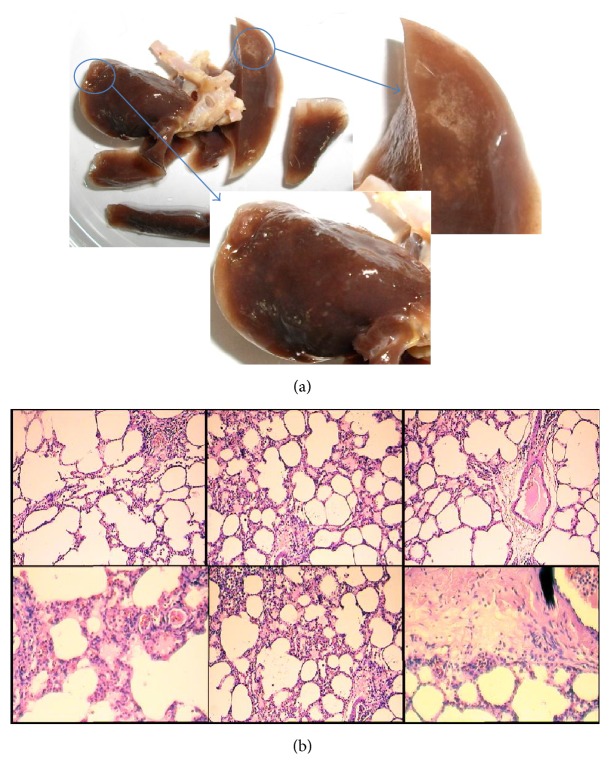
Representative of inflamed rat lung (a) and histological sections from the inflamed rat lung (b).

Figure 6(a) shows that the changes in lung tissue structure and yellow pale to white color spots were found in both lobs of lung and no solid tumors were observed. The micrograph of lung tissue sections showed that there were spots where the blood cells were found outside of the vasculature (Figure 6(b)) and the endothelial cells were found within these spots. These spots might correspond to angiogenesis. Perls' Prussian blue with hematoxylin counterstain method confirmed that hemin was abundantly accumulated in those of infiltrative points.

## 4. Discussion

Inflammation caused by an accumulation of red blood cells and leucocytes outside the blood vessel of the tissue which leads to angiogenesis was traditionally studied by biochemical methods called “analyzing frozen cross sections” of tumor tissues by using high-resolution microscopy. Indeed this method provides evidence of biological events. However, situations and conditions in such studies are forthcoming arising that requires dynamic imaging of live animals. Therefore, this study examined the potential use of ferrioxamine as a targeted MRI contrast agent for visualizing and determining any inflammation related to angiogenesis formation in temporally immunosuppressive rats xenografted with human small cell lung carcinoma. As clearly showed ferrioxamine exhibits cytotoxicity against small lung carcinoma cells 4 times lesser than desferrioxamine. Allain et al. reported that ferrioxamine was eliminated from healthy subjects by fast decline (*t*_1/2_ = 2.4 h) and slow decline (*t*_1/2_ = 5.9 h) processes [26]. The same authors also determined the renal clearances which is equal to 516 mL h^-1 ^kg^−1^ in healthy subjects. Moreover, desferrioxamine is used as iron chelator in clinical level [27]. Thus the injection of ferrioxamine in animals and human should be relatively safe. As a matter of fact, ferrioxamine is an efficient T1-contrast agent that can increase the sensitivity of proton imaging in a range of submicromolar concentration. The concentration of ferrioxamine at a given tissue was obtained by measuring the density of images by ROI technique. The density data were calculated in ferrioxamine concentration using (4). The wash-in and wash-out of a given tissue can be determined (see Figure 2). The results of this study also indicated that a majority (80%) of ferrioxamine was found in the vasculature system while a fraction (20%) resided in the tissues. Not found in normal rats are the bright spots present in both lung lobes of the xenografted rats. The distribution of bright spots was seen using 3D fat-suppressed MIP projections. These bright spots might be caused by the interaction of ferrioxamine with hemin liberated from the old red blood cell lysis or from a high vasculature density (lots of blood vessels over a small area). However, the same results were not observed when similar series of experiments were performed using Gd-DTPA (data were presented in poster session at WMIC 2012). It should be noted that ferrioxamine has higher permeability than Gd-DTPA suggesting that an enhancement of T1w-images should primarily have the result of the hemin accumulation at the inflammation points. This was consistent with histological results.

This study gets successful to develop immunocompromised rat model of lung cancer and demonstrates that by using hemin as biomarker and ferrioxamine as targeted MR probe. We can examine the inflammation sites that correlate very well with histological study using high-resolution microscope. However, the preparation procedure of the immune-compromised rat model of lung cancer can also be applied for another type of cancer strictly with respect to the protocol as follows. The animal model required for such study should initially be immune suppressed by 4 consecutive days of intraperitoneal injection of cyclosporin A (200 mg·kg^−1^) which caused a depletion of immunity prior to cancer cell implantation. We had found that these immune-compromised rats did not reject the implanted human cancer cell. Then the rat's immune system recovered when the immunosuppression was stopped after implantation. The recovery of rat immunity should decrease the growth and invasion rat of cancer cells. As can be seen the cancer tissue appearance was taken about 4 to 6 months later. During this period, the human cancer cells should progressively prepare their microenvironments by interacting with the host cells resulting in a cancer stromal structure for adhering and caused leakage of red blood cells and leucocytes to outside the blood vessels of a tissue following inflammation. By following the procedure of this study, seven out of nine rats (77%) have developed lung inflammation within 3 months. It should be noted that the cancer tissues obtained at the end of series of experiment were covered by a very dense and elastic tissue that distinguishes human cancer tissue from those of rats. The primary disadvantage of the temporally immunocompromised rat models is it time-lag. It is a model of cancer that very closely resembles developments that take place in the human body.

## 5. Conclusion

Overall the results of this study suggest that ferrioxamine is a suitable molecular probe for MR imaging of hemin accumulation that caused the inflammation of tissues, particularly at the very early stages of cancer.
